# A single episode of high intensity sound inhibits long-term potentiation in the hippocampus of rats

**DOI:** 10.1038/s41598-017-14624-1

**Published:** 2017-10-26

**Authors:** J. L. de Deus, A. O. S. Cunha, A. L. Terzian, L. B. Resstel, L. L. K. Elias, J. Antunes-Rodrigues, S. S. Almeida, R. M. Leão

**Affiliations:** 1 Department of Physiology, FMRP, University of São Paulo, Ribeirão Preto-SP, Brazil; 2 Department of Pharmacology, FMRP, University of São Paulo, Ribeirão Preto-SP, Brazil; 3 Department of Psychology, FFCLRP, University of São Paulo, Ribeirão Preto-SP, Brazil

## Abstract

Exposure to loud sounds has become increasingly common. The most common consequences of loud sound exposure are deafness and tinnitus, but emotional and cognitive problems are also associated with loud sound exposure. Loud sounds can activate the hipothalamic-pituitary-adrenal axis resulting in the secretion of corticosterone, which affects hippocampal synaptic plasticity. Previously we have shown that long-term exposure to short episodes of high intensity sound inhibited hippocampal long-term potentiation (LTP) without affecting spatial learning and memory. Here we aimed to study the impact of short term loud sound exposure on hippocampal synaptic plasticity and function. We found that a single minute of 110 dB sound inhibits hippocampal Schaffer-CA1 LTP for 24 hours. This effect did not occur with an 80-dB sound exposure, was not correlated with corticosterone secretion and was also observed in the perforant-dentate gyrus synapse. We found that despite the deficit in the LTP these animals presented normal spatial learning and memory and fear conditioning. We conclude that a single episode of high-intensity sound impairs hippocampal LTP, without impairing memory and learning. Our results show that the hippocampus is very responsive to loud sounds which can have a potential, but not yet identified, impact on its function.

## Introduction

Loud noises are a constant presence in the modern urban world. Not only sound pollution from urban traffic, but also the exposure to loud sounds from occupational (musicians and military personnel, for instance) and recreational (headphones, rock concerts) sources are increasingly common, especially among younger individuals^1^. The most common problems associated with loud noise exposure are hearing loss, tinnitus (hearing of a constant non-existent sound) and hyperacusia, which are commonly expressed as comorbities^2–4^. Besides the auditory deficits, intense noise exposure produces deleterious mental and systemic effects^5–7^. For instance, children chronically exposed to airport noise present reading comprehension and recognition memory deficits^8^, and even in children exposed to an environment with less intense noises presented similar cognitive deficits^9^. Additionally, traumatic blast also can have deleterious emotional and cognitive consequences in humans and rats^10–12^.

The hippocampus is a region traditionally implicated in the formation of declarative and spatial memories, and presents several forms of synaptic plasticity, including long-term potentiation and depression (LTP and LTD, respectively). These forms of synaptic plasticity are considered to be a form of synaptic memory, reinforcing the more efficient synapses in generating action potentials in the post-synaptic neurons^13^. In the hippocampus, the most studied form of synaptic plasticity is the associative NMDA receptor-dependent LTP of the Schaffer-CA1 pathway^14^. The hippocampus is functionally connected to the central auditory pathway indirectly from the frontomedial cortex, insula and amygdala and also connects back to the amygdala and auditory cortex via the enthorrinal cortex^15^. This pathway is implicated in the formation of long-term auditory memories^16^, and auditory cues can be used in the formation of spatial memories^17^. Recently it has been demonstrated that hippocampal place cells can be activated by an auditory dimension task^18^.

The hippocampal function is affected by different patterns of sound stimulation or sound deprivation. Chronic moderate sound exposure (80 dB, 2 hours/ day for 3–6 weeks) impairs spatial memory in mice and increases oxidative damage and tau phosphorylation in the hippocampus^19,20^. Acute traumatic noise (106 dB, 30 minutes) alters place cell activity in the hippocampus^21^. On the other hand, daily exposure to music (60 dB, 6 hours per day for 21 days) enhanced learning performance and increased BDNF expression in the hippocampus,^22^ a neurotrophin implicated in LTP generation and memory formation^23^. Sound deprivation by reversible conductive hearing loss reversibly impairs learning and memory in old rats and decreases the levels of cholinergic markers in the hippocampus^24^. Additionally, noise-induced hearing loss induces deficits in spatial memory and hippocampal neurogenesis in mice^25^. Even pre-natal exposition to loud noises can have deleterious effects on the hippocampi’s offspring^26,27^. These evidences show that the acoustic environment can impact the function of the hippocampal network affecting the animal’s behavior.

High intensity sounds can be considered stressors, and exposure to single periods of high intense noise (105 dB-30 minutes) activates corticotropin-releasing hormone neurons in the hypothalamic paraventricular nucleus, and stimulate the secretion of plasmatic corticosterone^28–30^. These effects were intensity-dependent and were abolished by lesions of the medial geniculate body, the auditory thalamic nucleus^28,30^. Corticosteroids are known to strongly affect hippocampal LTP, which is depressed by high levels of this hormone^31,32^. So, it is plausible that at least some of the effects of high intensity sound seen on the hippocampus are mediated by the secretion of corticosterone in response to sound stress.

Recently we reported that long-term exposure to short episodes of high intensity sound stimulation (120 dB, one minute twice a day for 10 days) inhibits the LTP in the Schaffer-CA1 pathway in the hippocampus of rats, but did not affect spatial learning and memory in the Morris Water Maze (MWM)^33^. These results showed that short episodes of traumatic sound for a long period affects the generation of long-term synaptic plasticity in the hippocampus, but curiously did not affect spatial learning. In the present work we studied the effects of a single episode of high-intensity sound stimulation on the hippocampal Schaffer-CA1 LTP and its dependency on the secretion of corticosterone. We found that a single episode of high-intensity sound stimulation is able to decrease LTP in a 24-hour time window, without affecting spatial learning and memory in the MWM. Additionally, we did not find correlation with the effect of high-intensity sound and corticosterone secretion, strongly suggesting that high-intensity sound itself, but not stress, is affecting LTP.

## Results

### A single sound stimulus of 110 dB is sufficient to inhibit LTP and PTP in the Schaffer-CA1 pathway

Our previous observation was that long-term exposure (10 days) to short (1 minute) episodes of high intensity sound (120 dB, 1 minute, twice a day) inhibits LTP in the Schaffer-CA1 synapse after 1 week of the end of the protocol^33^. Our goal in this work is to investigate if a single episode of high intensity noise exposure is able to alter LTP.

**Figure 1:**
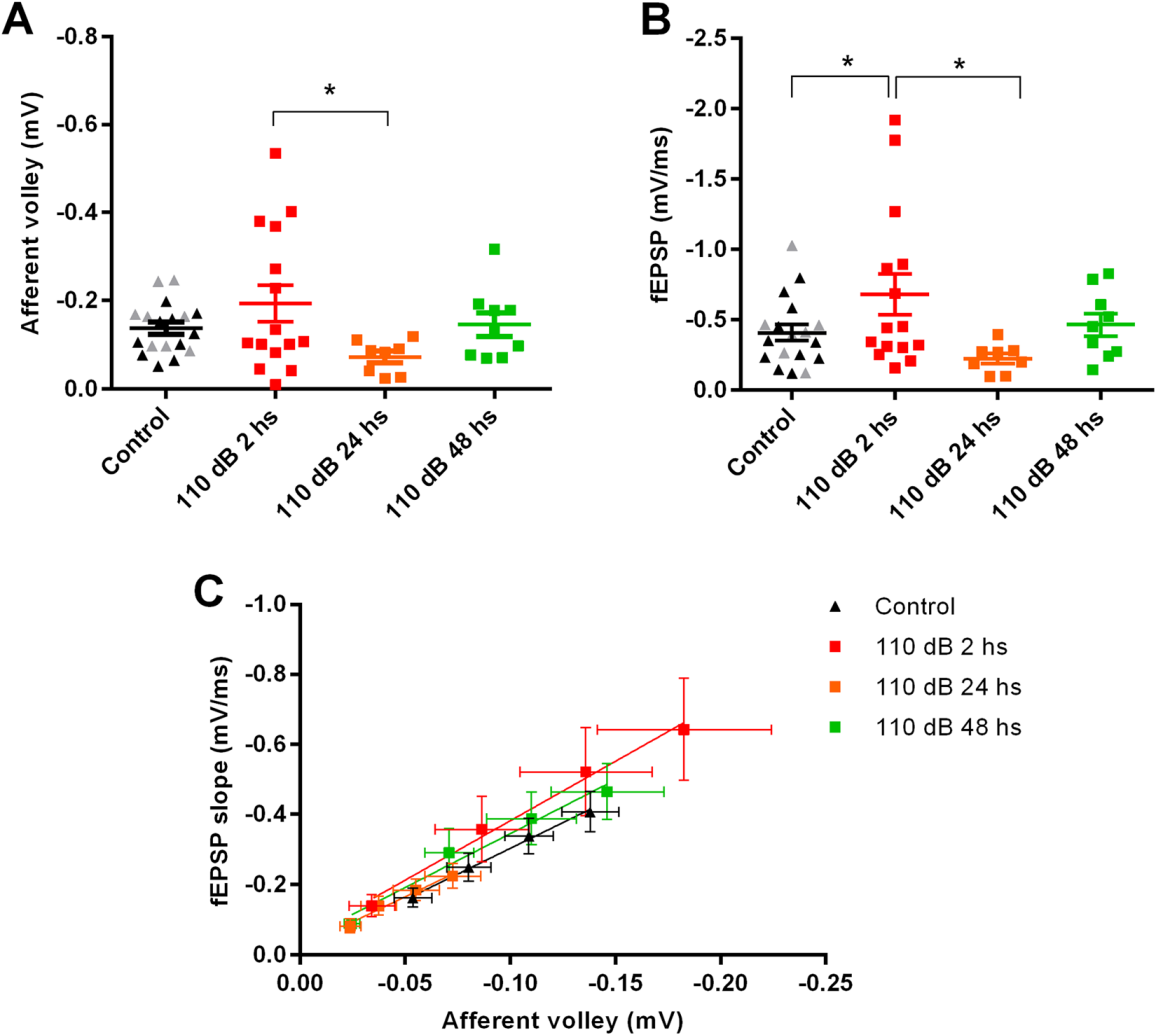
Effects of high-intensity sound stimulation on the neurotransmission on the Schaffer-CA1 synapses. (**A**) Maximum afferent volley amplitudes from control animals, and animals sacrificed 2, 24 and 48 hours after 110 dB sound stimulation. (**B**) fEPSPs slopes from control animals, and animals sacrificed 2, 24 and 48 hours after 110 dB sound stimulation. In the control groups, the dark symbols represent naïve animals, and the grey symbols, sham animals. (**C**) Correlation of fEPSP slope with afferent volley amplitude. Lines represent the fitting of linear functions. *p < 0.05.

We compared neurotransmission and LTP in the hippocampus of rats exposed to 110 dB noise 2, 24 and 48 hours after the acoustic stimulation. Stimulation of the Schaffer-CA1 produce fEPSPs with similar maximum slopes (P = 0.5813) and afferent volleys (P = 0.6309) in naïve and sham animals (P > 0.05, unpaired t- test), so we pooled the data together. Comparing all groups, we found that both the afferent volleys and the fEPSP slopes were significantly different (compared at the maximum stimulation; p < 0.05, one-way ANOVA). The fEPSP slope from the 2 hour animals was significantly bigger than the fEPSP slope from the control and 24 hours group (F(3, 46) = 3.086; P = 0.0363) and the afferent volley (F (3, 46) = 4.52; P = 0.0242; Fischer´s LSD; Fig. 1A,B). We constructed an input-output curve plotting the fEPSP slopes versus the afferent volleys (Fig. 1C) in response to crescent stimulation intensities and found no differences among the slopes of the relationships (F = 0.609, P = 0.6272).

**Figure 2:**
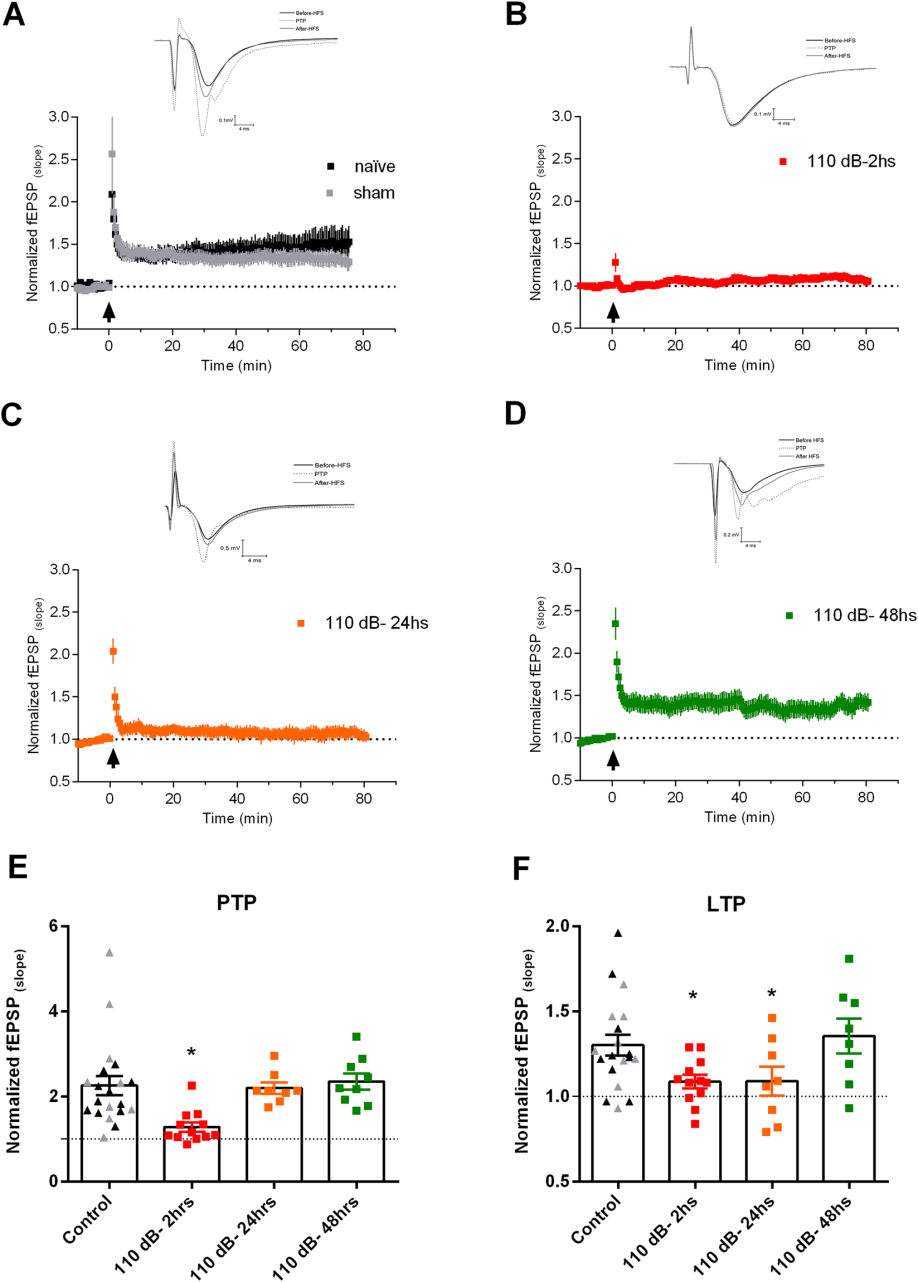
LTP inhibition by high-intensity sound stimulation. (**A**) Normalized fEPSP slopes before and after HFS (arrow) from the Schaffer-CA1 synapse of naïve and sham animals. Representative examples are shown in the inset. (**B**) Normalized fEPSP slopes before and after HFS (arrow) from the Schaffer-CA1 synapse of animals sacrificed 2 hours after 110 dB sound exposure. Representative examples are shown in the inset. (**C**) Normalized fEPSP slopes before and after HFS (arrow) from the Schaffer-CA1 synapse of animals sacrificed 24 hours after 110 dB sound exposure. Representative examples are shown in the inset. (**D**) Normalized fEPSP slopes before and after HFS (arrow) from the Schaffer-CA1 synapse of animals sacrificed 48 hours after 110 dB sound exposure. Representative examples are shown in the inset. (**E**) Summary of the PTP. (**F**) Summary of the LTP. *p < 0.05.

We then tested if LTP was affected by high intensity sound exposure. In these experiments, 100 Hz stimulation of the Schaffer-collateral fibers induced a strong post-tetanic potentiation similar in both sham and naïve animals (naïve: 2.0 ± 0.13; sham: 2.56 ± 0.46; n = 11 and 9, respectively. P = 0.224, unpaired t-test) followed by a long-term potentiation (that lasted at least 80 minutes after the protocol) which was not different between the naïve and sham groups (control: 1.34 ± 0.09; Sham: 1.29 ± 0.7; n = 11 and 9, respectively. P = 0.68, unpaired t-test). These data were then pooled together as a control group. On the other hand, both PTP and LTP from animals submitted to 110 dB of sound stimulation were strongly inhibited, comparing to control, after 2 hours of the stimulus (Fig. 2; PTP: 1.28 ± 0.11, F(3, 43) = 5.47, P = 0.0027; one-way ANOVA. P < 0.001, Fischer LSD test; LTP: 1.09 ± 0.04, F(3, 43) = 3.572, P = 0.0215; one-way ANOVA; P < 0.05 Fischer LSD test). In slices obtained from animals after 24 hours the sound stimulation PTP returned to values similar to control (Fig. 2; 2.2 ± 0.13; n = 8; P > 0.05 Fischer´s LSD test) but LTP was still significantly smaller than control (1.09 ± 0.08; n = 8; P < 0.05, Fischer´s LSD test). However, after 48 hours both PTP and LTP returned to values similar to control levels (PTP: 2.35 ± 0.19; LTP: 1.35 ± 0.1; n = 9. P > 0.05. Fischer´s LSD test). We conclude that 1 minute of 110 dB sound stimulation is sufficient to reversibly inhibit LTP and PTP in the Schaffer/CA1 pathway.

### A singe episode of 110 dB sound stimulation does not affect paired-pulse facilitation

LTP and PTP were reduced by a single episode of 1 minute of 110 dB sound stimulation. We found that fEPSP slope was also bigger 2 hours after sound stimulation, along with the afferent volley. Although LTP in the Schaffer-CA1 is mainly post-synaptic^34^, PTP is a pre-synaptic form of short-term plasticity that lasts several seconds and is dependent on a rise in the residual calcium which can increase release probability, vesicle pool size and quantal size^35–40^. On the other hand, paired-pulse facilitation (PPF) lasts milliseconds and is dependent on residual calcium accumulation in the terminal generating an increase in release probability^40^. Alterations in pre-synaptic parameters, like release probability or vesicle pool size, would affect the ratio of the second to the first EPSP in the PPF. In order to know if the changes in PTP were caused by changes in the basic pre-synaptic parameters, or by specific mechanisms of PTP, we measured PPF evoked by 2 stimuli delivered at short intervals (50, 150, 250, 350, 450 and 550 ms).

**Figure 3:**
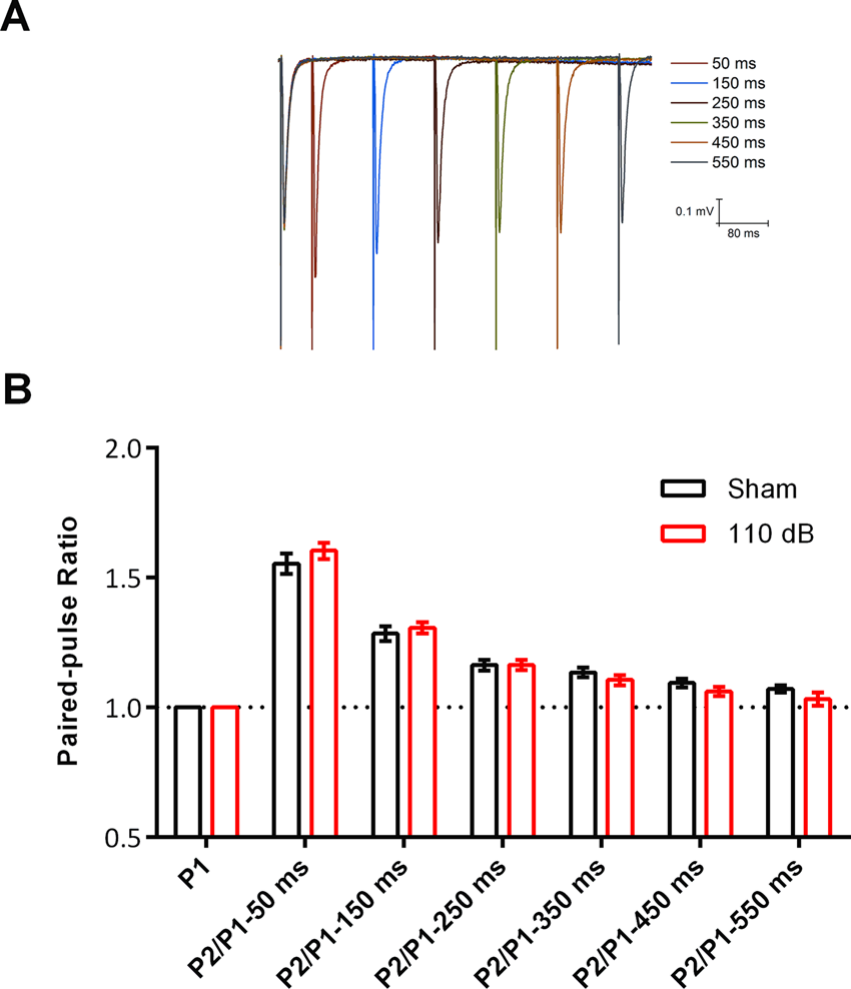
Effect of 110 dB sound exposure on short-term plasticity. (**A**) Pairs of fEPSPs delivered 50, 150, 250, 350, 450, and 550 ms apart. (**B**) Paired Pulse Ratios (PPR) of the fEPSPs delivered at the different intervals. N = 7, each group. Data represent mean ± SEM.

We found no changes in PPF in all inter-stimulus intervals of the Schaffer-CA1 synapses in slices from animals subjected to one minute 110 dB stimulation (Fig. 3: 50 ms: sham: 1.554 ± 0.03; 150 ms: 1.284 ± 0.02; 250 ms: 1.163 ± 0.02; 350 ms: 1.134 ± 0.01; 450 ms: 1.094 ± 0.01; 550 ms: 1.071 ± 0.01. 110 dB stimulation: 50 ms: 1.604 ± 0.03; 150 ms: 1.307 ± 0.02; 250 ms: 1.163 ± 0.01; 350 ms: 1.10 ± 0.01; 450 ms: 1.061 ± 0.01; 550 ms: 1.032 ± 0.02; p > 0.05 for all intervals, multiple t- tests corrected for multiple comparison with the Holm-Šídák test). The decay of the PPF was assessed fitting a single decay exponential function to the data, and the time constants were not considered different (K = 0.008 ± 0.001, sham and 0.007 ± 0.001, 110 db, resulting in taus of 124 and 143 ms respectively. P > 0.05). Therefore, we conclude that the basic pre-synaptic mechanisms of vesicle exocytosis are not affected by our high intensity sound stimulation protocol, and that the changes in PTP probably reflects specific changes in the mechanisms of PTP.

### A sound stimulus of 80 dB does not inhibit LTP

**Figure 4:**
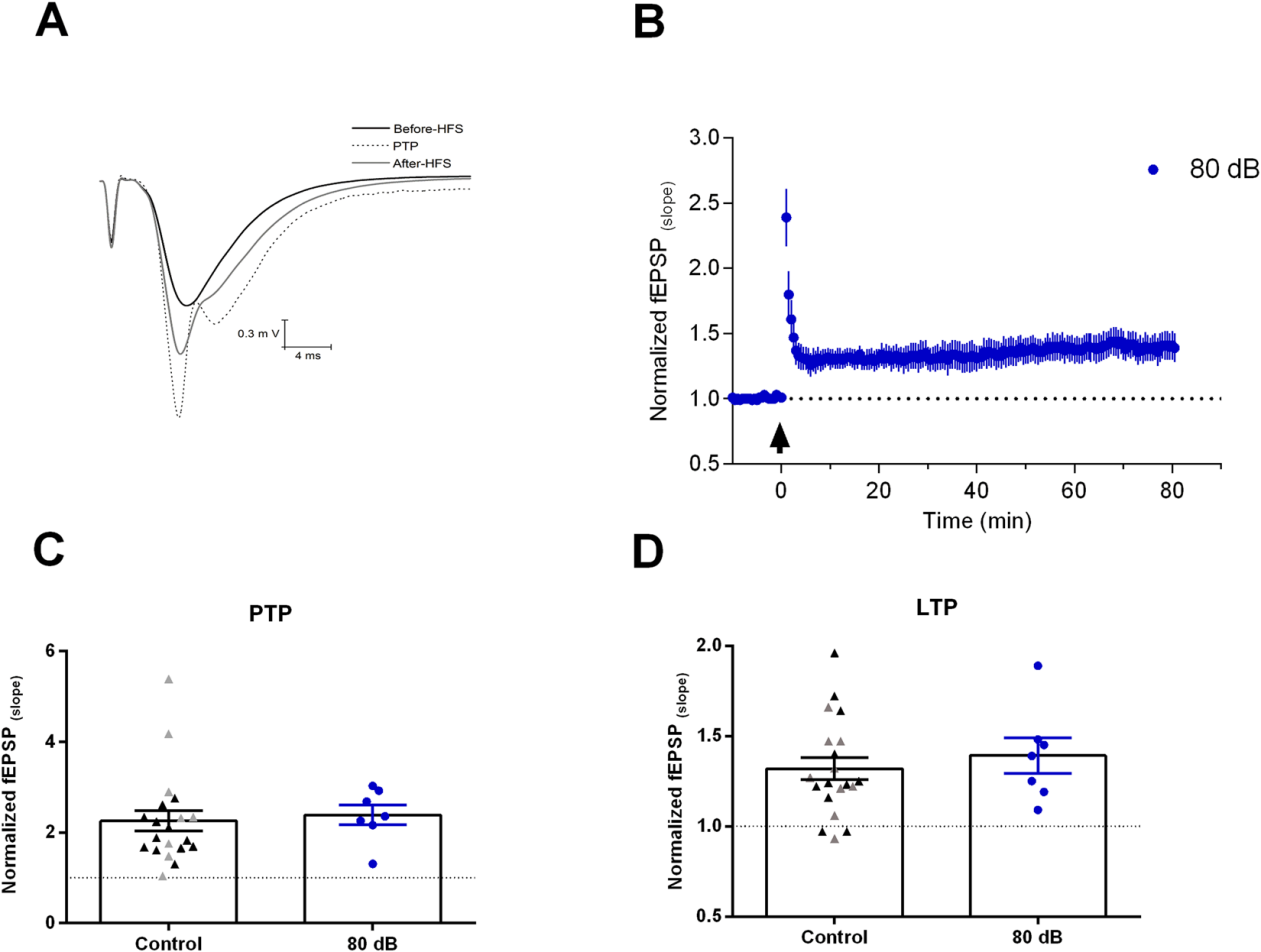
Sound stimulus of 80 dB does not inhibit LTP. (**A**) Representative examples of fEPSPs before and after HFS (PTP and LTP) from an animal exposed to 80 dB sound stimulation. (**B**) Normalized fEPSP slopes before and after HFS (arrow), in the Schaffer-CA1 synapse of animals sacrificed 2 hours after 80 dB sound exposure. (**C**) Summary of PTP. (**D**) Summary of LTP. Controls as in Fig. 2. Data represent mean ± SEM.

We then tested if the LTP inhibition was related to the sound intensity. We subjected the animals to a sound stimulation with a non-traumatic moderated sound level of 80 dB, and sacrificed the animals 2 hours later. We found no difference in the maximum fEPSP slope and afferent volley from control animals (afferent volley. Control: −0.14 ± 0.013 mV; 80 dB: −0.16 ± 0.08 mV; P = 0.513, unpaired t-test; fEPSP. Control: −0.41 ± 0.06 mV/ms; 80 dB: −0.62 ± 0.09 mV/ms; P = 0.07, unpaired t-test). After the 100 Hz stimulus, both LTP and PTP developed normally in the slices from these animals, with no different from the control group (PTP 80 dB: 2.38 ± 0.2; P = 0.74; LTP 80 dB: 1.39 ± 0.09; P = 0.54, n = 7; Fig. 4). We conclude that the effects of sound stimulation on hippocampal LTP depends on sound intensity.

### High-intensity sound stimulation inhibits the LTP in the perforant pathway-dentate gyrus synapses

**Figure 5:**
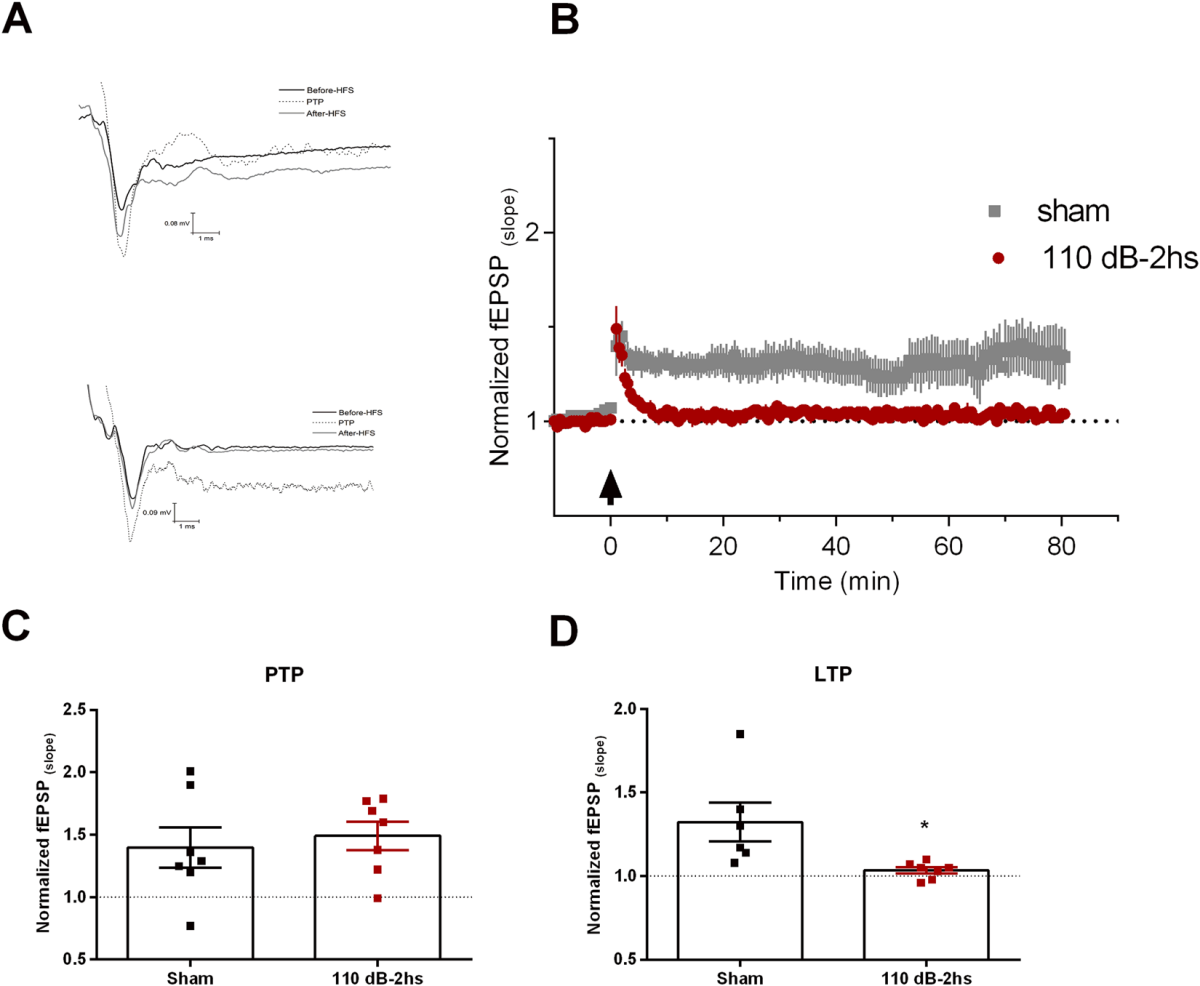
High intensity sound inhibits LTP in the PF-DG pathway. (**A**) Representative examples of fEPSPs before and after HFS (PTP and LTP) from an animal exposed to sham stimulation and an animal exposed to 110 dB sound stimulation. (**B**) Normalized fEPSP slopes before and after HFS (arrow), in the PF-DG synapses of animals sacrificed 2 hours after 110 dB sound exposure. (**C**) Summary of PTP. (**D**) Summary of LTP. *p < 0.05.

Was the effect of high intensity sound specific for the Schaffer-CA1 synapse? In order to answer this question, we recorded LTP from the first synapse in the hippocampal tri-synaptic circuit, the perforant fiber-dentate gyrus (PF-DG) synapses. Stimulation of the medial PF generated fEPSPs in the external molecular layer DG, which were similar in both sham and stimulated group (fPESP slope: Sham, −0.31 ± 0.05 mV/ms. Stimulated, −0.32 ± 0.03 mV/ms. P > 0.05, unpaired t-test; n = 7 for both groups). We also did not find differences in the afferent volleys of both groups (Sham, −0.05 ± 0.010 mV. Stimulated, −0.05 ± 0.007 mV. P > 0.05, t-test; n = 7 for both groups). Similarly, to what was observed in the Schaffer-CA1 synapse, we found a significant smaller LTP in the slices from animals subjected to 110 dB sound stimulation (Sham: 1.3 ± 0.11; Stimulated: 1.03 ± 0.01; P = 0.022; unpaired t-test; n = 6 and 7, respectively), but no effect on the PTP (Sham: 1.39 ± 0.16, Stimulated: 1.49 ± 0.11; P = 0.64; unpaired t-test. n = 7 for both groups) (Fig. 5). These findings show that high intensity sound stimulation effects are not restricted to the Schaffer-CA1 pathway.

### High intensity sound stimulation does not increase the plasmatic corticosterone levels

**Figure 6:**
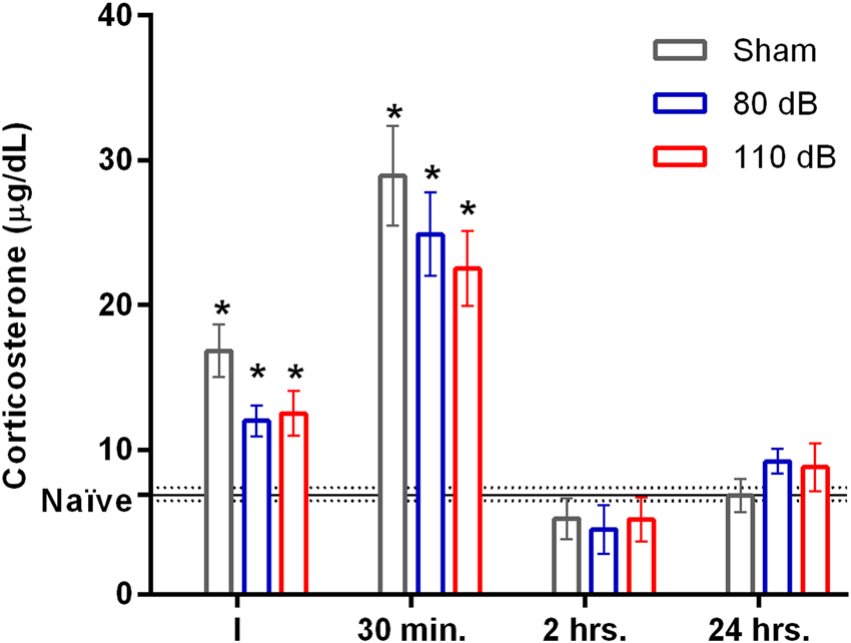
Plasmatic corticosterone. Plasmatic corticosterone levels in naïve, sham, 80 dB and 100 dB animal groups, sacrificed immediately (~30 seconds; I), 30 minutes, 2 and 24 hours after sham or sound stimulation. The horizontal lines are the mean (solid line) ± SEM (dashed lines) of the corticosterone levels of the naïve group. *p < 0.05 compared with the naïve group.

Because increased plasmatic corticosterone induced by stress affects the hippocampal LTP^31,32^ and high intensity noise increases plasmatic corticosterone^28,30^, we decided to investigate if the effects of high-intensity sound stimulation could be correlated to the activation of the hypothalamus-pituitary-adrenal axis (HPA), by measuring the secretion of corticosterone after sound stimulation. We compared the plasmatic corticosterone in animals subjected to sham, 110 and 80 dB sound stimulation, and compared to control animals (6.9 ± 0.55 µg/dl), in different periods after stimulation. Surprisingly, we found that plasmatic corticosterone increased in all 3 groups immediately (sham: 16.8 ± 1.8 µg/dl; 110 dB: 12.5 µg/dl; 80 dB: 12.0 ± 1.0 µg/dl, F(3, 16) = 9.38, P = 0.0008, One-way ANOVA) and 30 minutes after stimulation (sham: 28.9 ± 0.5 µg/dl; 110 dB: 22.5 ± 2.6 µg/dl; 80 dB: 24.9 ± 2.9 µg/dl, F(3, 16) = 3.71, P = 0.0001, One-way-ANOVA) and but returned to normal (control) levels 2 and 24 hours after stimulation (Fig. 6). Because the plasmatic levels of corticosterone were similar in all groups F(2, 12) = 1.35, P = 0.29, (Two-way ANOVA), but the effect on LTP was restricted on the animals subjected to high intensity sound stimulation, we concluded that its effect on LTP is related to the intensity of the sound and not to an increase of plasmatic corticosterone. In this analysis, the interaction of sound stimulus versus time was not statistically significant.

### High-intensity sound stimulation does not affect spatial memory and learning

Hippocampal LTP has been demonstrated to be relevant for spatial learning and memory^41,42^, and the MWM is a test that evaluates spatial learning and memory which has been widely used to evaluate the effects of hippocampal synaptic plasticity^43^. Therefore, we tested the performance in the MWM of animals subjected to 110 dB of noise and compared with animals from the naïve, sham stimulated and noise stimulated with 80 dB noise groups.

**Figure 7:**
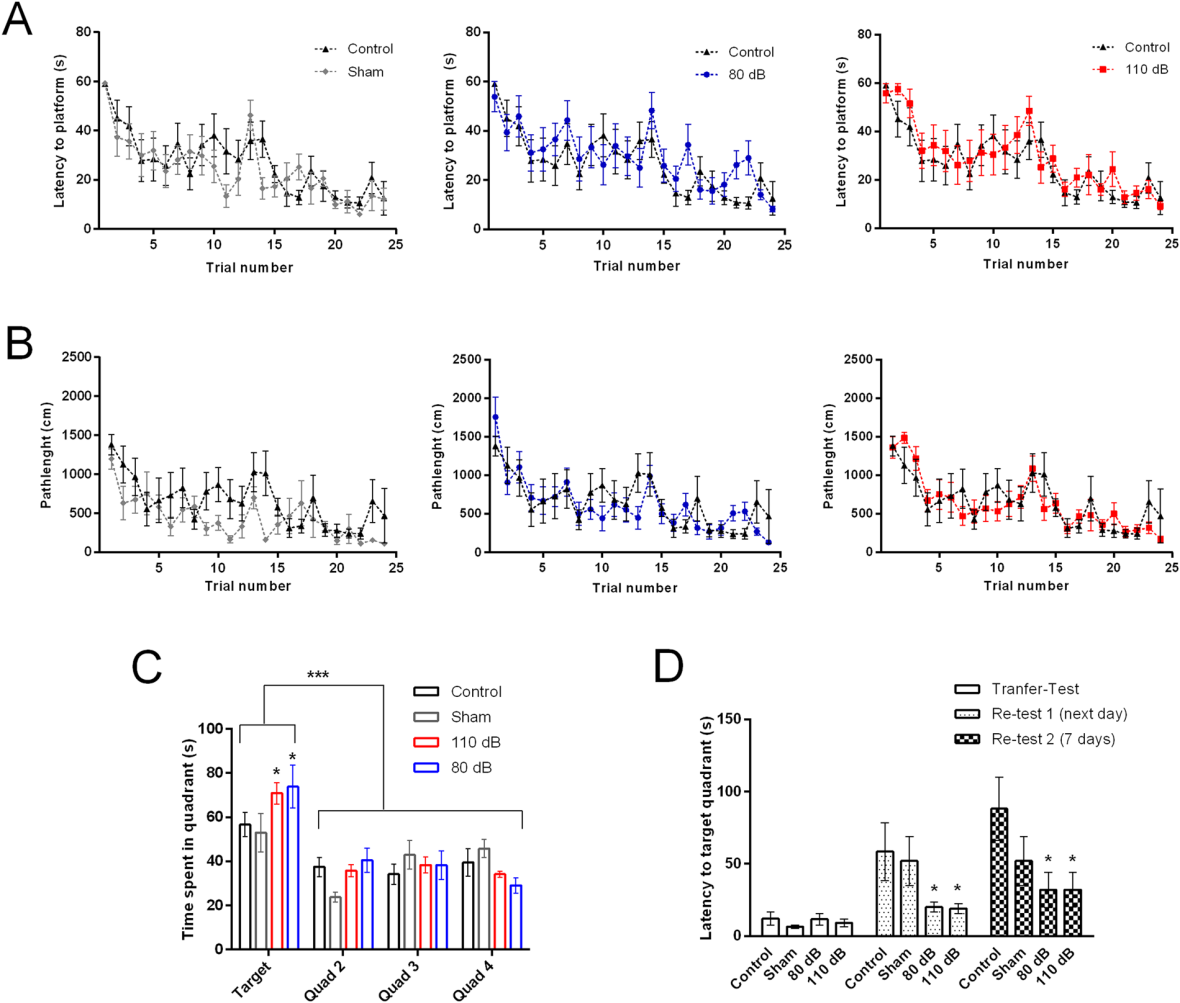
Spatial learning and memory in the MWM. (**A**) Latency to find the platform of animals from the Sham, 80 dB and 110 dB groups in comparison with naïve animals. (**B**) Mean pathlengths of animals during training trials. (**C**) Time spent in each quadrant during the probe trial without platform. (**D**) Mean escape latencies to platform during probe trial and re-tests. During probe trial, with removed platform, escape latencies refer to the first time the tracer identified the animal in the location where the platform was previously placed. *p < 0.05. ***p < 0.005. n = 5–8 per group.

As expected, latencies to find the hidden platform decreased with trials F(23, 23) = 14.19; P < 0.001; 2-way ANOVA, but all groups had learned to find the platform with similar escape latencies, with no difference between the experimental groups and the control group with F(3, 23) = 1.1; P = 0.54, two-way ANOVA. We did not observe differences in the stimulus versus trial interaction (p > 0.05). Similarly, the distance travelled to find the target quadrant did not differ among groups, F(3, 23) = 1.27; P = 0.30 (Fig. 7B). However when we performed a transfer-test without the platform after the last trial, we found that all groups spent more time in target quadrant F(3, 69) = 12.90; P < 0.001, but the 110 dB and 80 dB groups spent significantly more time in the target quadrant than the control and sham groups F(3, 23) = 44.24; P < 0.001 (Fischer´s LSD test t, P < 0.05) (Fig. 7C). When we analyzed the time spent in the target quadrant in blocks of 45 seconds we found that all groups had similar patterns of occupation of the target quadrant. Animals spent more time in the target in the first 45–90 seconds, and then spent significantly more time in the other quadrants; F(3, 72) = 18.03 P < 0.05, for quadrant occupation and F(3, 24) = 1.311; P = 0.29 for group of animals (Fischer´s LSD test, not shown), so there is no detectable increase in perseverance in the sound exposed group.

When we performed re-tests 24 hours and 7 days after the last trial to identify any memory retention deficit, we found significant differences in the probe trials, with the animals taking more time to find the target quadrant, 1 and 7 days later F(2, 69) = 12.08; P < 0.0001; two-way ANOVA, but interestingly, the animals subjected to 80 or 110 dB sound presented significantly shorter latencies to find the target quadrant F(3, 69) = 4.95; P = 0.0036; two-way ANOVA (p < 0.05. Fischer´s LSD as post-test; Fig. 7D). No differences were observed in the stimulus versus quadrants interaction (p > 0.05). We conclude that the inhibition of hippocampal LTP by acute 110 dB sound exposure, did not affect spatial learning and memory in rats, similarly to what we found in response to long term sound stimulation^33^. Curiously, sound presentation seemed to improve memory retention.

### High-intensity sound stimulation does not affect cued fear conditioning

**Figure 8:**
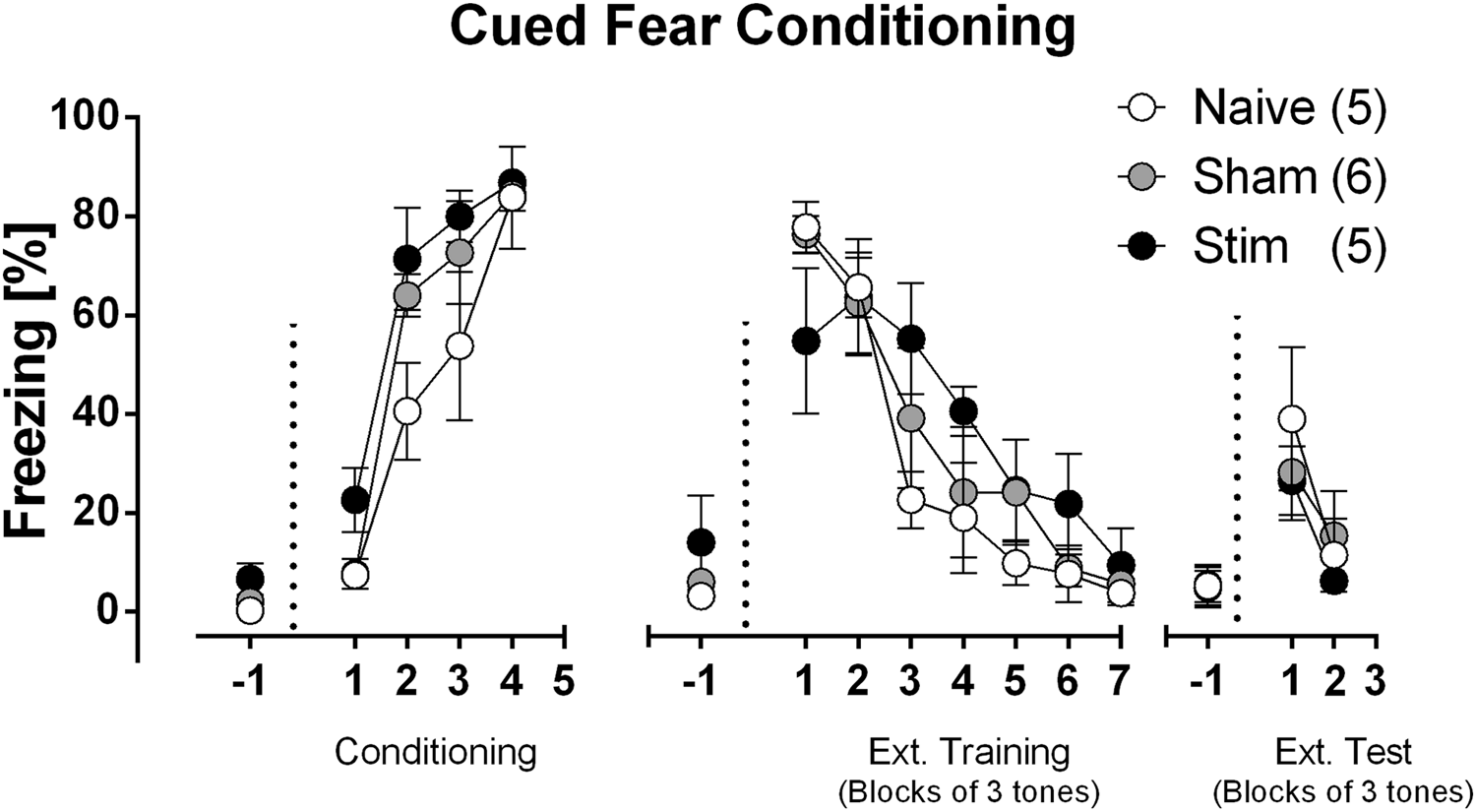
Cued fear conditioning test. Effects of 110 dB acoustic stimulation on cued fear conditioning. On Conditioning phase, the sound cued was paired with 4-shock exposure. During Extinction phase (Ext. Training; 24 h after Conditioning), 21 tones were presented and are graphically expressed in blocks of 3-tones. After additional 24 h, test for extinction learning (Ext. Test) was performed. Ten tones were presented and are graphically expressed in blocks of 3-tones. Basal freezing was scores for 2 minutes, before tone presentation. Data is expressed as mean ± SEM. Statistical analyses performed: Two-way ANOVA followed by Bonferroni’s post hoc test.

In order to know if the inhibited LTP after high intensity sound was able to affect other types of hippocampal-dependent memory we tested the rats for the cued fear conditioning protocol, a type of associative memory which has a hippocampal component^44^. During the cued fear conditioning protocol, all groups (naïve, sham and 110 dB) showed similar freezing levels during conditioning (Time: F(3, 39 = 1.07), P = 0.39; Treatment F(2, 13) = 3.67, P = 0.054), extinction training (Time: F(6, 78) = 40.5, P < 0.001; Treatment: F(2, 13) = 0.44, P = 0.65; = 1.97, P = 0.04) and in the test of extinction. (Time: F(2, 26) = 30.44, P < 0.001; Treatment: F(2, 13) = 0.35, P < 0.70) (Fig. 8). During all three phases, all groups showed significant differences in freezing behavior within each session, but no overall difference was observed between groups.

## Discussion

In this work we investigated the LTP-stress-memory triad in rats subjected to high-intensity noise stimulation. We showed that a single episode of high intensity sound (110 dB) is able to inhibit LTP in the Schaffer-CA1 synapse in the hippocampus, from 2 to 24 hours after sound exposure. This effect depends on sound level, is not correlated with corticosterone secretion, could be observed in other hippocampal glutamatergic synapse, the perforant pathway-dentate gyrus, and does not affect spatial learning and memory assessed in the MWM and cued fear conditioning.

Although it has been demonstrated that exposition to loud noises can alter hippocampal function^21,45^, its effect on hippocampal synaptic plasticity was reported only for long-term (7–10 days) sound exposure^27,33^. Interestingly, Barzegar and colleagues^27^ had shown that pre-natal exposure to sound (1 to 4 hours to 95 dB broadband noise for 7 days) compromised hippocampal LTP and performance on the MWM, but they found a concomitant increase in plasma corticosterone levels both in the mother and offspring. On the other hand, the LTP inhibition observed by us was not correlated with corticosterone secretion. We also found that LTP was inhibited even 10 days after the end of the long-term sound exposure protocol^33^ while the LTP inhibition by the acute exposure to sound reverted in 48 hours after the single protocol of sound exposure. Altogether these data show that both long-term and single high-intensity sound exposure can alter hippocampal function and synaptic plasticity, although differences exist in the chronic and acute effects.

We also found that, different from the observed with the long-term protocol, the fEPSP slope and afferent volleys were altered after sound stimulation. We found significantly bigger fEPSP slopes and afferent volleys in the 110 dB/2-hour group when compared to the other groups. On the other hand, the input-output relationship when normalized by the afferent volley was not different in all groups. These data suggest that the excitability of the afferent axons can be increased by loud sound exposure, resulting in more recruited afferent fibers generating larger fEPSPs. The glutamatergic release probability is not affected because the relationship afferent volley and fEPSP is similar in control and 110 dB and because there was no difference in the paired-pulse ratio after loud sound exposure. Additionally, because the relationship afferent-volley and fEPSP was similar in all groups, we do not believe that the LTP inhibition is caused by a saturation of the LTP by already potentiated synapses after sound exposure.

We also found a significant inhibition of PTP 2 hours after sound stimulation which reverted to control values 24 hours after sound stimulation. In the long-term protocol we observed a decrease in the PTP, which lasted 10 days after the end of the protocol. PTP is a short–term plasticity process which is attributed to increase in pre-synaptic residual calcium after the tetanic train, causing an increase in the neurotransmitter release probability (P_r_), quantal size (q) and in the readily releasable pool (RRP) of vesicles^35–39^. Because we did not see differences in the PPR, the fast calcium buffering system of these synapses are probably not affected by the sound stimulation, but the high-capacity buffering system, which is relevant for PTP could be altered. Additionally, the effects on PTP could be linked to the processes triggered by calcium influx, or on a reduced size of the vesicle pool, which might not be detected by the PPR protocol. Because LTP was inhibited 24 hours after sound stimulation, but PTP was back to control values, we believe that the mechanisms producing the inhibition of LTP and PTP are probably distinct.

In our previous work^33^ we hypothesized that the inhibition of LTP by the long term high-intensity noise exposure could be a response to a potentiated neurotransmission and/or LTP in the first synapses in the tri-synaptic hippocampal circuit. To test this hypothesis in the acute paradigm we studied the LTP in the perforant pathway-dentate gyrus synapse. We found similar maximum fEPSPs slopes and afferent volley amplitudes in the sham and 110 dB groups, and like in the Schaffer-CA1 synapse, an inhibition of the LTP in the animals subjected to 110 dB sound. We conclude that the inhibition of the LTP in the Schaffer-CA1 is not a response to an increased neurotransmission or enhanced LTP at upstream synapses. Additionally, we can conclude that the effect of high intensity sound stimulation on LTP is not restricted to the Schaffer-CA1 synapse.

It is well known that loud sounds are stressors and promote the activation of the HPA axis and release of corticosterone^25,29^, which is a suppressor of hippocampal LTP^31,32^. Thus, we tested the hypothesis that the sound-induced inhibition of LTP was related to corticosterone secretion during the exposure to the high-intensity sound protocol. In fact, we found an increase in corticosterone secretion immediately and at 30 minutes after high intensity sound exposure. However, we found similar increases in plasma corticosterone in both sham and 80 dB groups, which did not present an inhibition of LTP. Therefore, we concluded that the effect of the high-intensity sound on the LTP is related to the intensity of the sound and not a consequence of corticosterone secretion in response to acoustic stress. But, nevertheless we cannot rule out a complex participation of corticosterone in conjunction with other factor dependent on the sound intensity. We will investigate this in more detail further.

Because of the postulated relationship of LTP and spatial learning we tested the ability of the animals subjected to high intensity sound to perform in the MWM, a test traditionally used to evaluate the effects of hippocampal synaptic plasticity^41–43^. We previously found^33^ that rats subjected with the chronic high-intensity sound protocol performed normally in the MWM, despite having an inhibited LTP. We hypothesized that in the long-term exposure, the brain adapted to the deficit in LTP resulting in normal spatial memory and learning. However, we found that even with a diminished LTP after a single episode of high intensity sound stimulation, the rats performed similarly in the MWM than the rats in the control, sham and 80 dB groups. In the retests we found similar times to find the platform across all groups. We concluded that even with an impaired LTP, animals subjected to a single episode of high-intensity sound stimulation presented normal spatial learning and memory assessed by the performance in the MWM. A dissociation of spatial memory and learning, assessed using the MWM, and hippocampal LTP was also seen in animals with specific hippocampal genetic deletions of NMDA receptors subunits^46,47^ which presented reduced or absent LTP in the Schaffer-CA1 synapse, but presented normal performance in the MWM. However, it is likely as previously observed in hippocampal NDMA receptor–knock-out mice that other learning processes not tested are impaired by the decreased LTP^46,47^, or that the decrease in LTP we observe is not sufficient to impact spatial learning. Differently to our results, Liu and collegues^25^ found that animals subjected to noise trauma performed worse in the MWM, 3 months after sound exposure. But their results might be more related to the subsequent hearing loss than the sound exposure itself. Similarly, Manikandan and collegues^48^ also found deficits in spatial memory measured with radial maze after a chronic exposure (30 days, 4 hours per day) of high-intensity noise (100 dB), which were correlated with an increased plasma corticosterone. However, the hearing status of these animals was not assessed. These data are in contrast with our present and previous results^33^ which showed no visible deficits in spatial learning and memory.

Interestingly in the immediate re-test without the platform the rats exposed to noise (80 and 110 dB) spent more time in the target quadrant than sham and control animals, and presented shorter latencies to find the target quadrant in the later re-tests, suggesting an improvement of memory retention by sound stimulation, independent of the intensity.

Finally, we tested the effect of high intensity noise on cued fear conditioning. Due to the auditory stimulus characteristics, we decided to use tone as the CS, in order to observe if any alteration would be detected in this task after the stimulus. Similarly, to the MWM, we found that high-intensity stimulus does not alter aversive learning and memory, which are encoded by LTP in the hippocampus and other regions^49,50^. In cued fear conditioning, LTP processes are associated with amygdaloidal nuclei and projections from and to other regions including the hippocampal CA1 region^44,51,52^. Additionally, CS presentation alone is necessary and sufficient to trigger extinction of conditioned fear, which depends on hippocampus activity and NMDA receptor dependent LTP^53,54^. Our results showed that the impaired hippocampal LTP induced by high-intensity sound stimulation, is not sufficient to impact fear conditioning learning and extinction.

Because high intensity sound inhibited LTP *in vitro* but did not affect hippocampal dependent learning and memory, it is possible that *in vivo*, the hippocampal synapses are able to produce LTP or it can be compensated by LTP by other forebrain synapses^46^. It is interesting to note that the response to HFS was very variable, and although the average LTP in the slices from animals submitted to high intensity sound was very small, we could find slices which the neurotransmission was potentiated, while in other we found even depression after HFS, suggesting that not all synapses are equally affected by high-intensity sound. Another hypothesis for the inhibition of the LTP is a change in rules of LTP, an effect called metaplasticity^55^.

These changes in hippocampal synaptic plasticity induced by high-intensity sound might be important for homeostatic control of hippocampal excitability, while allowing normal function of the hippocampus. Interestingly, we found that in rats genetically susceptible to audiogenic seizures^56^, our long-term sound exposure protocol, which in these animals lead to limbic epileptic seizures, did not inhibit LTP^33^ suggesting that this effect could be important for the prevention of hippocampal seizures. Further experiments will be done to understand the mechanisms of the inhibitory effect of high intensity sound on hippocampal LTP.

In conclusion we showed that a single episode of high intensity sounds has a fast effect on the hippocampus, inhibiting LTP for 24 hours, an effect not correlated with the activation of the HPA axis by stress. However, this effect was unable to affect spatial navigation learning and memory assessed in the MWM, as well cued fear conditioning. Nevertheless, our results show that even a brief exposure to a high-intensity sound can have a profound impact on hippocampal synaptic plasticity, and very likely on animal’s hippocampal function.

## Methods

All experimental protocols involving animals were designed according to rules for animal research from the National Council for Animal Experimentation Control (CONCEA#006/2015) and approved by the Commission for Ethics in Animal Experimentation (CETEA) at the University of São Paulo in Ribeirão Preto.

### **Animals**

Male Wistar rats (60–70 days old) were kept in Plexiglas cages (four animals per cage), with food and water ad libitum and 12-h dark/light cycle (lights on at 7:00 a.m.) and controlled temperature (22 °C) at the Animal Housing Facility of the Department of Physiology, School of Medicine of Ribeirão Preto, University of São Paulo. The rats were divided in four groups: naïve rats (taken directly from their cages), sham (rats manipulated equally as the rats submitted to the acoustic stimulus protocol, but with no sound stimulation), rats submitted to acoustic stimulus of 110 dB (high-intensity sound stimulation) and rats submitted to acoustic stimulus of 80 dB.

### **Sound stimulation**

Rats were placed in an acrylic, acoustically isolated arena (height: 32 cm, diameter: 30 cm), located inside a sound proof chamber (45 × 45 × 40 cm), with 2 loudspeakers placed on the top of the arena, where, after one minute of acclimation, they were submitted to a 110 dB or an 80-dB noise stimulus (a digitally modified recording of a doorbell, spanning frequencies from 3 to 15 kHz^57^, with one minute duration. After stimulation the animals were kept in the cage for one more minute, and returned to their home cages. Sham animals were placed in the arena for 3 minutes. Ambient noise inside the acoustic chamber was around 55 dB. The sound intensity at the arena interior was checked and calibrated regularly with a decibelimeter. Some animals presented symptoms of mesencephalic seizures during the sound stimulation^33^ and were not used in this study.

### **Preparation of Hippocampal slices**

After 2, 24 and 48 hours of acoustic stimulation, animals were anesthetized with isofluorane and decapitated. Brains were rapidly removed and placed in an ice-cold solution containing (mM): 87 NaCl, 2.5 KCl, 25 NaHCO_3_, 1.25 NaH_2_PO_4_, 75 Sucrose, 25 Glucose, 0.2 CaCl_2_, 7 MgCl_2_, bubbled with 95% O_2_ and 5% CO_2_. The brain was glued with cyanoacrylate glue to a support, placed inside the cutting chamber of a vibratome and cut in 400 µm transversal slices containing the dorsal hippocampus in the same solution. Sections of the hippocampus were dissected out from the slices using ophthalmic scissors and micro-tweezers and placed in artificial cerebro-spinal fluid (aCSF) solution containing (mM): 125 NaCl, 2.8 KCl, 1.25 NaH_2_PO_4_, 26 NaHCO_3_, 10 Glucose, 2 CaCl_2_, 1 MgCl_2_. Slices were left to rest for at least two hours before use (one hour at 34–35 °C and at least one hour in room temperature) and continuously bubbled with carbogenic mixture (95% O_2_ and 5% CO_2_).

### **Field potential recordings and LTP induction**

Electrophysiological recordings were performed at controlled temperature of 32–34 °C using an inline heater (Warner Instruments, USA) with a Multiclamp 700B amplifier (Molecular Devices, USA) connected to a Digidata 1440 A AC/DC interface (Molecular Devices, USA). Slices were placed in a recording chamber with continuous superfusion of aCSF (1 mL/min) bubbled with carbogenic mixture, and kept in place with a nylon thread in a platinum frame. A stainless steel bipolar concentric microelectrode (FHC - Bowdoin, Maine, USA), connected to a Master-9 voltage stimulator (A.M.P.I., Israel), was placed on the Schaffer-collaterals fibers or perforant pathway. Field excitatory post-synaptic potentials (fEPSPs) were recorded at CA1 *stratum radiatum* and external molecular layer of dentate gyrus with borosilicate glass microelectrodes (G85150T, Warner Instruments, USA) filled with aCSF, with tip resistances of 1–2 MΩ and connected to the amplifier probe through a silver wire covered with AgCl. For recording of fEPSPs in the dentate gyrus, picrotoxin (100 μM) was added to the aCSF.

First, we performed input-output curves, where voltage was gradually increased by 10 V increments, until population spikes were observed in the fEPSP. In order to obtain a baseline response, we stimulated the Schaffer fibers and perforant pathway (set at 50% of the maximum response) at 0.03 Hz for 25 minutes. We considered for analysis fEPSPs with big amplitudes in relation to the afferent volley amplitudes. After a stable baseline, LTP was induced on the Schaffer-collaterals fibers with 3 trains of high frequency stimulation (HFS) at 100 Hz of 1 second duration (3 seconds of inter-train interval) and on the perforant pathway with 3 trains of HFS at 100 Hz of 1 second duration (20 seconds of inter-train interval). HFS trains of 100 Hz induced a short post-tetanic potentiation (PTP) followed by LTP of the fEPSPs that lasted at least 80 minutes.

Signals were acquired at 100 kHz and low pass filtered at 3 kHz (Bessel, 8-pole). All data were acquired with pClamp 10.2 software (Molecular Devices, USA).

### **Hormone extraction and radioimmunoassay**

Plasmatic hormonal extractions and radioimmunoassay of corticosterone was performed from 25 μL of plasma extracted with 1 mL of ethanol as described previously^58,59^. Animals were killed by decapitation and blood collected at 4 distinct times: immediately (less than one minute), 30 minutes, 2 and 24 hours after sham or sound stimulation (110 dB or 80 dB). A control group (naïve) was used as a baseline for comparison. All blood samples were collected between 10–12 am.

### **Spatial navigation memory test (Morris Water Maze)**

The Morris Water Maze (MWM) consisted of a circular pool painted in black (1.40 m diameter × 50 cm, depth) filled with water at 23 °C, with a black platform (9 cm – diameter) placed in one of the virtual four quadrants. Visual clues were placed on the walls surrounding the pool. Two hours after sound stimulation, we started the training sessions of MWM test. The platform was always placed in the same quadrant (target quadrant), and the starting quadrant was chosen randomly by the software. Rats were placed at the border of starting quadrant (excluded the target quadrant). We used the same sequence of quadrants for all animals. They could explore the pool for 90 seconds or until they found the platform. Between trials, the animals could rest at the platform for 30 seconds and placed on another quadrant for the next trial. The platform remained at the same position during the entire training trials. The rats were subjected for 12 daily trials for 2 days. At the end of the second day of training trials the platform was removed from the pool for performing the probe trial (25^th^ trial- transfer-test). In this case, with removed platform, escape latencies were estimated as the first time the program identified the animal in the location where the platform was previously placed. After 24 hours (P2) and one week (P3) of the last trial, the rats were released in the pool in the quadrant opposite to the target quadrant (with the platform present) in order to evaluate the memorization. The rat was allowed to explore the pool for 180 s.

Trials were recorded using a video camera (SA-3 Tracker, USA) placed on top of the pool and animals were tracked with the Ethovision tracking system (Noldus Information Technology, The Netherlands) which calculated the latencies to the hidden platform and distances travelled.

### **Cued fear conditioning**

For cued fear condition we used two different boxes: context A (23 × 20 × 21 cm; white walls, except for a transparent plexiglass; one grid floor containing 23 stainless steel rods, 2 mm in diameter, spaced 1.0 cm apart, and wired to generate footshocks) and context B (23 × 20 × 21 cm, black and white striped walls, except for a transparent plexiglass one, and white floor) (Insight, Ribeirão Preto, Brazil).

Two hours after sound stimulation, the animals were placed in context A for 2 minutes before they received foot-shocks (4 shocks, 0.85 mA, 1 s; unconditioned stimulus; US) paired with an auditory cue (30 s, 1 kHz, 70 dB; conditioned stimulus; CS). Twenty-four hours later, during the Extinction training, animals were placed in context B, placed in a different room, while 21 tones were presented without foot-shock. On the following day, 10 tones were presented to test extinction learning, in context B. Freezing behavior was evaluated during all three phases, being characterized by total lack of movements except for those necessary to breathing^60^.

### **Data analysis and statistics**

Data were analysed with Clampfit 10.2. For analysis, the recordings were low-pass filtered offline (500 Hz) and the slopes of the fEPSPs were fitted with a linear function. Data from each experiment were normalized relative to its baseline. LTP was quantified as the average of the fEPSP slopes in the last 35 minutes of the 80 minutes post-induction recording period. Input-output curves for fEPSPs and afferent volleys were fitted with a linear function and their slopes compared. Comparison across groups was done using unpaired t-tests, multiple t-tests corrected for multiple comparisons with the Holm-Šídák test or one-way ANOVA with a LSD Fischer’s post-test.

Data from Morris Water Maze and cued fear conditioning were analysed using two way-ANOVAs with repeated and non-repeated measures with LSD Fischer´s or Bonferroni post-test. Data are shown as mean ± SEM and for analysis we used GraphPad Prism 5.0 and Origin Lab software. Significance level was set at p ≤ 0.05.
